# Serine Supplementation Alleviates Doxorubicin-Induced Oxidative Damage in Skeletal Muscle of Mice

**DOI:** 10.3389/fphys.2021.727093

**Published:** 2021-09-09

**Authors:** Jingqing Chen, Xihong Zhou, Hai Jia, Zhenlong Wu

**Affiliations:** ^1^State Key Laboratory of Animal Nutrition, China Agricultural University, Beijing, China; ^2^Laboratory Animal Center of the Academy of Military Medical Sciences, Beijing, China; ^3^Key Laboratory of Agro-ecological Processes in Subtropical Region, Institute of Subtropical Agriculture, Chinese Academy of Sciences, Changsha, China

**Keywords:** doxorubicin, mitochondria, oxidative stress, serine, skeletal muscle

## Abstract

Muscle weakness affects physical activity and quality of life of patients. Serine, a nutritionally non-essential amino acid has been reported to enhance protein synthesis and implicate in biosynthesis of multiple bioactive molecules. It remains unknown whether it can protect mice against oxidative stress-induced muscles weakness. This study was conducted to test the hypothesis that serine administration alleviates doxorubicin-induced oxidative damage in skeletal muscle of mice. Mice pre-treated with or without serine were intraperitoneally injected with either doxorubicin or equal volume of saline. Reactive oxygen species (ROS) accumulation, activity of antioxidant enzymes, oxidation product of protein, DNA, and lipid, activity of mitochondrial complex, and protein level of nuclear-factor-erythroid-2-related factor 2 (NRF2)/constitutive-androstane-receptor (CAR) signaling in skeletal muscle of mice were determined. Compared with the control, doxorubicin exposure led to oxidative damage as shown by increased ROS accumulation, decreased activity of antioxidant enzymes, and enhanced oxidative product of protein, DNA, and lipid in the skeletal muscle of mice. These effects of doxorubicin were associated with increased activity of complex I and reduced glutathione. Interestingly, doxorubicin-induced oxidative damage was alleviated by serine administration. Further study showed that the beneficial effect of serine was associated with enhanced NRF2/CAR signaling. Our result showed that serine attenuated doxorubicin-induced muscle weakness in mice. Serine supplementation might be a nutritional strategy to improve the function of skeletal muscle in patients exposed to doxorubicin.

## Introduction

Doxorubicin is a widely-used chemotherapeutic agent for a variety of tumors in patients (Smuder et al., 2011). Application of doxorubicin has been reported to be associated with muscles weakness and fatigue in patients (Yamamoto et al., 2008; van Norren et al., 2009). These side effects affect quality of life and physical activity, and result in restricted treatment efficacy and poor prognosis. The myotoxicity of doxorubicin is mainly mediated by oxidative damage of proteins, lipid, and DNA (Schwartz, 2000), due to increased production of reactive oxygen species (ROS) in mitochondria of skeleton muscle following doxorubicin administration (Smuder et al., 2011; Gilliam et al., 2013). However, therapeutic strategies to alleviate doxorubicin-induced skeleton muscle dysfunction are not available yet.

The glutathione (GSH) is one of the well-known endogenous antioxidants with ability to scavenge ROS, including reactive species, therefore maintaining intracellular homeostasis (Gaucher et al., 2018). Depletion of intracellular GSH has been reported to be associated with ROS accumulation and oxidative damage (Gaucher et al., 2018), which can be reversed by overexpression of γ-glutamylcysteine ligase (Gcl), a rate-limiting enzyme for GSH synthesis (Meister and Anderson, 1983). Importantly, clinical studies show that patients receiving doxorubicin treatment have a decreased intracellular GSH level and skeleton muscle dysfunction, as compared with the non-treatment individuals (Min et al., 2015; Songbo et al., 2019), indicating that boosting GSH content might be a potentially therapeutic target in ameliorating doxorubicin-induced muscle dysfunction.

L-Serine (serine) has traditionally been considered as a nutritionally nonessential amino acid due to its *de novo* synthesis. Serine plays an essential role in various cellular processes, such as one carbon unit metabolism, synthesis of glycine and cysteine, as well as cellular survival (Kalhan and Hanson, 2012; Metcalf et al., 2018). Offspring from dams fed with a serine deficient diet is vulnerable to oxidative stress (He et al., 2018), indicating a requirement for serine to exert an anti-oxidative capacity. Further studies show that the beneficial effect of serine on muscle weakness is associated with restoration of redox status in animals (Sim et al., 2015; Zhou et al., 2017a,b, 2018a; Cao et al., 2018; He et al., 2018) or various types of cells (Zhou et al., 2018b,c). In the present study, we hypothesized that serine pre-administration prevented doxorubicin-induced oxidative damage of muscle in mice. This hypothesis was based on the observation that doxorubicin exposure lead to a reduce cysteine, a substrate for synthesis of GSH in skeletal muscles (Fabris and MacLean, 2018), which might contribute to reduced GSH level and oxidative damage in the skeleton muscle and other tissues of humans and rodents. Furthermore, serine serves as a metabolic substrate for synthesis of glycine and cysteine, which can be used for GSH synthesis, catalyzed by Gcl, a rate-limiting enzyme (Kalhan and Hanson, 2012; Gaucher et al., 2018), therefore restoring intracellular redox homeostasis.

## Materials and Methods

### Animal and Diet

The experimental protocol was approved by the Protocol Management and Review Committee of the Institute of Subtropical Agriculture, the Chinese Academy of Science (Changsha, China). A total of 32 6-week-old male C57BL/6 mice were purchased from SLAC Laboratory Animal Central (Changsha, China), and raised in an environment with a temperature set at 22°C and a 12/12h light-dark cycle. Purified AIN93 diet as previous described (Reeves et al., 1993) and water were provided *ad libitum*. After a 7-day adaptive period, mice were randomly divided into four groups: CON (control), serine (orally administered with serine, 1.5gkg BW^−1^ day^−1^ for 4weeks), doxorubicin (20mgkg BW^−1^, i.p.), or Ser-Dox (serine supplementation, and then were subjected to doxorubicin treatment). The dose of doxorubicin used in the present study was based on previous study showing that doxorubicin administration results in oxidative damage in skeletal muscle of mice (Smuder et al., 2011; Kavazis et al., 2014; Min et al., 2015). Forty-eight hours after doxorubicin treatment, all the animals were euthanized by cervical dislocation; skeletal muscles from the upper hindlimb were collected for later analysis.

### Preparation of Whole-Muscle Lysates

For the preparation of whole-muscle lysates, skeletal muscles from the upper hindlimb were homogenized in ice-cold lysis buffer [1mM dithiothreitol (DTT), 10mM NaCl, 1.5mM MgCl_2_, 20mM 4-(2-hydroxyethyl)-1-piperazineethanesulfonic acid (HEPES), 20% glycerol, and 0.1% Triton X-100; pH 7.4] containing protease inhibitors. After a centrifugation at 12,000*g* for 15min at 4°C, the supernatant was collected, and the protein concentration was measured using a bicinchoninic acid assay. Mitochondria were isolated according to the method as previously described (Trounce et al., 1996). Briefly, the supernatant was centrifuged at 8,000*g* for 15min, and then was resuspended in isolation buffer (210mM mannitol, 1mM EGTA, 70mM sucrose, and 5mM HEPES) and recentrifuged at 8,000*g* for 15min. The resulting mitochondrial pellet was used for further analysis.

### Determination of the Oxidation Products

Reactive oxygen species was determined as previously described (McMillan and Quadrilatero, 2011). Briefly, whole-muscle lysates or mitochondrial pellets were incubated with 5μM 2',7'-dichlorodihydrofluorescein diacetate (DCFH-DA; Invitrogen, Carlsbad, CA, United States) for 20min at 37°C in the dark. The samples were analyzed using a microplate spectrofluorometer with an excitation and an emission wavelength of 490 and 535nm, respectively. Data were expressed as arbitrary units per milligram of protein. The superoxide radical contents were determined based on the methods as described (Azzi et al., 1975; Hong and Lee, 2009). Briefly, whole-muscle lysates were added to a reaction mixture containing 90mM succinate, 50mM phosphate buffer (pH 7.5), 30nM KCN, 150mM KCl, and 0.3mM cytochrome *c*, and mixed in an oscillator for 5min. The absorbance was measured at a wavelength of 550nm. The hydrogen peroxide contents in the tissues were measured based on protocol as previously described (Jiang et al., 1992; Hong and Lee, 2009). Briefly, whole-muscle lysates were mixed with 100mM sorbitol, 100μM xylenol orange, 250μM ammonium ferrus sulfate, and 25mM H_2_SO_4_. After incubation at 25°C for 30min, the absorbance at 560nm was measured.

### Measurements of Oxidative Damage

DNA was isolated from the skeletal muscles of the upper hindlimb using a DNA isolation kit obtained from Qiagen (Shanghai, China). The levels of 8-hydroxy-2'-deoxyguanosine (8-OHdG) were measured as previously described (Mecocci et al., 1999). The results were expressed as the ratio of nanomoles of 8-OHdG to nanomoles of 2-deoxyguanosine×10^5^. Whole-muscle lysates were used for the determination of malondialdehyde (MDA) and protein carbonyl by using specific enzyme-linked immunosorbent assay (ELISA) kits (NWK-MDA01 and NWK-PCK01, respectively; Northwest Life Science Specialties, Vancouver, WA, United States) according to the manufacturer’s instructions. The MDA concentration of samples was determined by measuring the absorption at 532nm. The protein carbonyl was determined by measuring the absorbance at 450nm. Lipids were extracted from the skeletal muscles of the upper hindlimb using Folch solution, and the level of 8-isoprostane was determined using an ELISA kit (NWK-ISO01, Northwest Life Science Specialties) according to the manufacturer’s instructions. The absorbance at 450nm was measured after the addition of an acid stop solution which causing a color change to yellow.

### Measurement of Mitochondrial Oxidative Markers

The activities of complex I (NADH ubiquinone reductase), complex II (succinate-coenzyme Q reductase), complex III (ubiquinol cytochrome c reductase), complex IV (cytochrome oxidase), and citrate synthase (CS) in the skeletal muscle were assessed spectrophotometrically using commercial Assay Kits (catalogue No BC0515, BC3230, BC3240, BC0945, and BC1064, respectively; Solarbio, Beijing, China) according to the manufacturer’s instructions. The absorbance was measured at 340nm for complex I, 605nm for complex II, 550nm for complex III, 550nm for complex IV, and 412nm for citrate synthase, respectively.

### Measurements of Antioxidant Enzyme

The activities of catalase (CAT), manganese superoxide dismutase (MnSOD), and copper-zinc superoxide dismutase (CuZnSOD) in the whole-muscle lysates were measured using commercial ELISA kits (NWK-CAT01 and NWK-SOD02, respectively; Northwest Life Science Specialties) according to the manufacturer’s instructions. CAT activity was measured by monitoring the consumption of the H_2_O_2_ substrate at 240nm. The activity measured using the original assay buffer reflects total superoxide dismutase (SOD) activity, whereas the activity measured in KCN-containing assay buffer is only due to MnSOD activity. The absorbance was measured at 560nm.

### Determination of the Intracellular Glutathione Antioxidant System

Reduced glutathione (GSH), oxidized glutathione (GSSG) content, and the activities of glutathione S-transferase (GST), glutathione reductase (GRD), and glutathione peroxidase (GPX) were determined using commercial ELISA kits (Item No 703002, 703302, 703202, and 703102, respectively; Cayman Chemical Company, Ann Arbor, Michigan, United States), according to the manufacturer’s instructions. GSH content was calculated by measurement of the absorbance of 5-thio-2-nitrobenzoic acid at 405–414nm. Determination of GSSG was performed by prior GSH derivatization with 2-vinylpyridine using the same kit. Total GST activity was determined by measuring the absorbance at 340nm after a conjugation of 1-chloro-2,4-dinitrobenzene with reduced glutathione. GRD activity was determined by measuring the absorbance at 340nm after oxidation of NADPH to NADP^+^. GPX activity was indirectly evaluated through a coupled reaction with GRD; the absorbance was measured at 340nm. Gcl activity was determined according to the method described in previous studies (White et al., 2003; Wu et al., 2009). Briefly, the skeletal muscles were homogenized in ice cold buffer (0.25M sucrose, 20mM Tris, 20mM boric acid, 1mM EDTA, 1mM l-serine, and pH 7.4), and a supernatant was obtained by centrifuging at 20,000*g* for 30min at 4°C. The supernatant was mixed with 100μl cysteine and 100μl reaction cocktail (40mM MgCl_2_, 2mM EDTA, 20mM boric acid, 2mM l-serine, 400mM Tris, 40mM ATP, and 40mM l-glutamic acid). After incubation for 30min at 37°C, 100ml of 200mM 5-sulfosalicylic acid was added, and the supernatant was collected by a centrifugation at 2,000*g* for 10min, which was used for the measurement of fluorescence intensities at an excitation wavelength of 485nm, and an emission wavelength of 538nm.

### qRT-PCR Analysis

RNA was isolated from mixed upper hindlimb using TRIzoL reagent (Invitrogen Corporation, Carlsbad, CA, United States). One microgram of RNA from each sample was reverse-transcribed to complementary DNA (cDNA) in 20-μl reactions (Qiagen, Germantown, MD, United States). The resulting cDNA was used for qPCR using the primers shown in Supplementary Table 1. Briefly, 1μl of the cDNA template was added to a total volume of 10μl reaction solution containing 5μl SYBR Green mix (Qiagen), 3μl deionized H_2_O, 0.2μl Rox, and 0.4μl of the forward and reverse primers. Real-time PCR was performed using the ABI-Prism 7500 Sequence Detection System (Applied Biosystems) as instructed by the manufacturer. All the genes were normalized to the housekeeping gene, and the relative differences in gene expression among the groups were determined using the comparative *C*_t_ value method (Livak and Schmittgen, 2001).

### Protein Qualification by the Wes Simple Western System

Protein expression extracted from samples of the skeletal muscles was qualified using the Wes Simple Western System (Proteinsimple, San Jose, CA, United States). Proteins were mixed with Proteinsimple mixed reagent and then loaded into Wes 25-well plates. Primary antibodies [β-actin, lamin B, constitutive-androstane-receptor (CAR), nuclear-factor-erythroid-2-related factor 2 (NRF2), and Keap1 (Kelch like ECH-associated protein 1), Abcam, Cambridge, MA, United States], secondary antibodies, and stacking gel and separation gel matrix were added according to the manufacturer’s instructions. Results were collected using the Protein Simple software (Proteinsimple).

### Treadmill Testing

Treadmill testing was performed as previously reported (Kadoguchi et al., 2015). In brief, after a 10-min adaption, the mouse was placed on the treadmill (Columbus Instruments, Columbus, OH, United States) set at 6m/min and 0° for 10min, after which, the inclination was set at 10° and the speed was gradually increased by 2m/min every 2min until the mouse reached exhaustion. Exhaustion was defined as spending at least 10s on the shocker plate without trying to re-engage the treadmill. Work was defined as the product of the vertical running distance to exhaustion and body weight.

### Statistical Analysis

Trial experiments or experiments done previously were used to determine sample size with adequate statistical power. Statistical analysis was performed by factorial ANOVA using a mixed procedure (PROCMIXED) with SAS software version 9.2 (SAS Institute Inc., Cary, NC, United States). All data were analyzed by a normality test, and variance was compared using a Bartlett’s test. Differences between means were determined by using the Duncan multiple-comparison test. For 2-factor ANOVA, the PROC general linear model was used, with l-serine and doxorubicin as main factors. Data are presented as means±SEM. A value of *p*<0.05 was considered significant.

## Results

### Effects of Serine Administration on Oxidative Damage in the Skeletal Muscles of Mice

**Figure 1:**
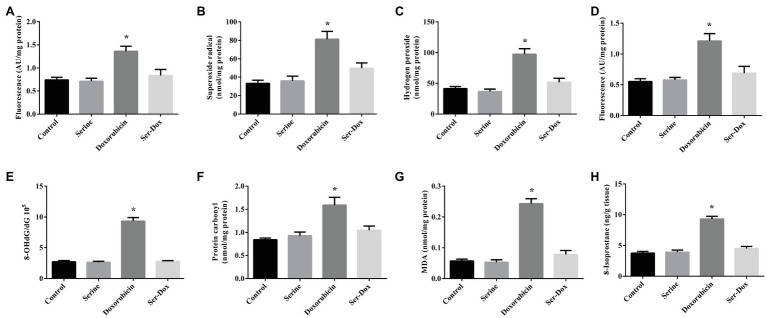
Oxidation product contents in the skeletal muscles of mice. ROS content **(A)**, superoxide radical **(B)**, and hydrogen peroxide **(C)** contents in the skeletal muscles; ROS content in mitochondria **(D)**. 8-OHdG **(E)**, protein carbonyl **(F)**, MDA **(G)**, and 8-isoprostane **(H)** contents in the skeletal muscles. Values are expressed as LSmean plus pooled SEM, *n*=8. ^*^Mean values were significantly different among groups (*p*<0.05). ROS, reactive oxygen species; 8-OHdG, 8-hydroxy-2'-deoxyguanosine; and MDA, malondialdehyde.

As shown in Figure 1, doxorubicin exposure led to increased ROS contents in both the cellular and mitochondria, as compared with the controls (Figures 1A,D). This effect of doxorubicin was attenuated by serine pre-administration. Further study showed that doxorubicin treatment resulted in elevated levels of superoxide radical and hydrogen peroxide in skeletal muscles, which were markedly reduced by serine administration (Figures 1B,C). Consistently, doxorubicin treatment led to oxidative damage to DNA, protein, and lipid, as shown by increased contents of 8-OHdG, protein carbonyl, as well as MDA and 8-isoprostane, respectively (Figures 1E–H). These deleterious effects of doxorubicin were remarkably prevented by serine administration, indicating a protective effect of serine on oxidative damage in skeletal muscles of doxorubicin-challenged mice.

### Effects of Serine Administration on Mitochondrial Respiratory Complex in the Skeletal Muscles of Mice

**Figure 2:**
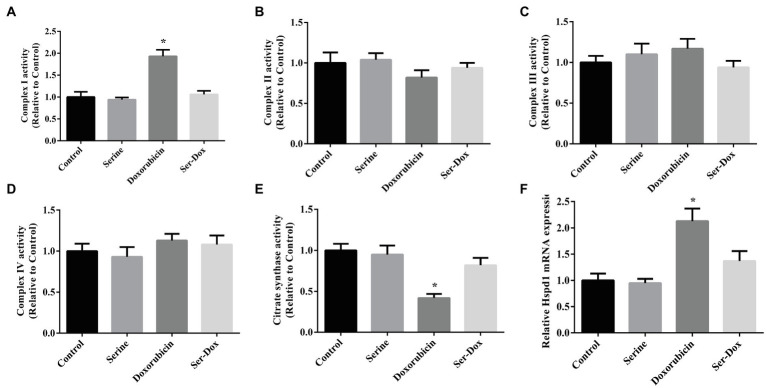
Mitochondrial respiratory complex in the skeletal muscles of mice. Activity of complex I **(A)**, complex II **(B)**, complex III **(C)**, complex IV **(D)**, citrate synthase **(E)**, and mRNA expression of Hspd1 **(F)** in the skeletal muscles. Values are expressed as LSmean plus pooled SEM, *n*=8. ^*^Mean values were significantly different among groups (*p*<0.05). Hspd1, heat shock protein 1.

As shown, doxorubicin treatment led to enhanced activity of complex I (Figure 2A), without affecting the activities of complex II, complex III, or complex IV in skeletal muscles (Figures 2B–D). The effect of doxorubicin on activity of complex I was prevented by serine administration. Also, we found that doxorubicin treatment led to decreased activity of citrate synthase (Figure 2E), while increasing mRNA level of Hspd1 (Figure 2F), as compared with the controls. These results indicated that serine mainly regulated activity of respiratory complex I in doxorubicin-challenged mice.

### Effects of Serine Administration on the Activities of Antioxidant Enzymes and Glutathione Content in the Skeletal Muscles of Mice

**Figure 3:**
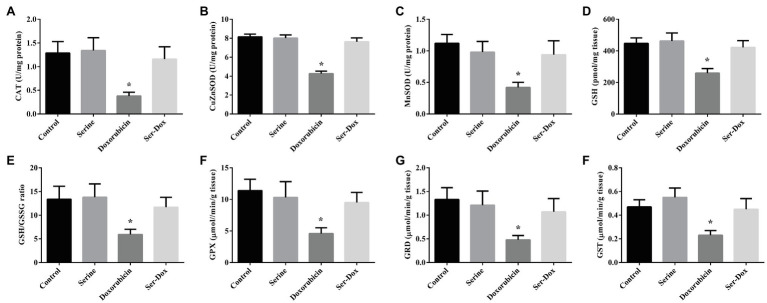
The activities of antioxidant enzymes and glutathione content in the skeletal muscles of mice. CAT **(A)**, CuZnSOD **(B)**, and MnSOD **(C)** activities in the skeletal muscles; GSH content **(D)**, GSH/GSSG ratio **(E)**, GPX **(F)**, GRD **(G)**, and GST **(H)** activities in the skeletal muscles. Values are expressed as LSmean plus pooled SEM, *n*=8. ^*^Mean values were significantly different among groups (*p*<0.05). CAT, catalase; MnSOD, manganese superoxide dismutase; CuZnSOD, copper-zinc superoxide dismutase; GSH, reduced glutathione; GSSG, oxidized glutathione; GST, glutathione S-transferase; GRD, glutathione reductase; and GPX, glutathione peroxidase.

Compared with the control, the activities of CAT, CuZnSOD, and MnSOD were significantly reduced following doxorubicin treatment, which were abrogated by serine pre-administration (Figures 3A–C). To explore an implication of non-enzymatic antioxidant system in our animal model, the activity of glutathione was measured. As indicated, GSH content, the ratio of GSH to GSSG, the activities of GPX, GRD, and GST were significantly reduced by doxorubicin treatment (Figures 3D–H). Interestingly, these alterations triggered by doxorubicin were abolished by serine pre-administration. In contrast, the mRNA levels for genes involved in the thioredoxin and peroxiredoxin system, including TRX, TRR, PRX1, and PRX3, were not affected by doxorubicin, serine, or doxorubicin plus serine treatment (Supplemental Figure 1), thus excluding an involvement of these genes in our animal model.

### Effects of Serine Administration on the Amino Acid Contents and GSH Synthesis in the Skeletal Muscles and Livers of Mice

Serine, as well as glycine which could be used for endogenous synthesis of serine, were measured in the skeletal muscle of mice. The contents of serine and glycine (Supplementary Figures 2A,B) increased significantly following doxorubicin treatment. However, orally serine administration had no effect on the amino acids changes in the skeletal muscles of the doxorubicin-treated mice. In addition, we determined serine and glycine in the liver and the contents were decreased significantly in the liver, which were significantly attenuated by the presence of serine (Supplementary Figures 2D,E). To explore whether the beneficial effect of serine supplementation was mediated by GSH synthesis in the muscle, we determined the content of cysteine (a key substrate for GSH synthesis) and activity of Gcl (an enzyme for GSH synthesis), and found that both of them were not affected by serine supplementation in doxorubicin-challenged mice (Supplementary Figures 2C, 3A). Considering that the liver is the largest organ and main source of GSH synthesis, we speculated that the increase of GSH content in the skeletal muscle might due to an elevated production of GSH in the liver, which was confirmed by both the content of substrates (glycine and cysteine), Gcl activity, and production of GSH in the liver tissues of doxorubicin-challenged mice (Supplementary Figures 2D–F, 3C,D).

### Effects of Serine Administration on NRF2/CAR Signaling in the Skeletal Muscles of Mice

**Figure 4:**
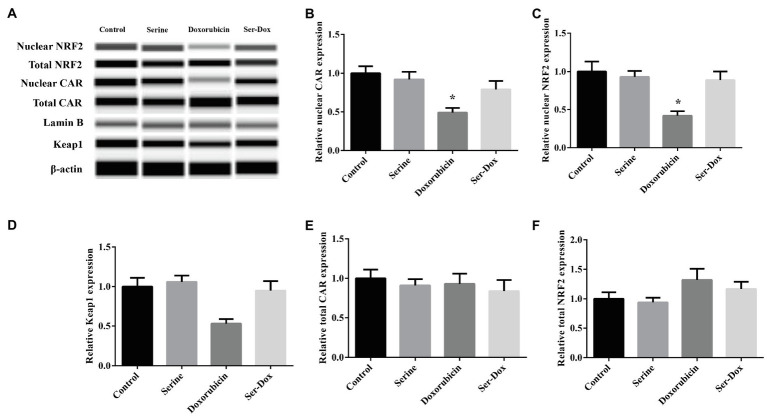
Effects of serine administration on NRF2/CAR signaling in the skeletal muscles of mice. Representative protein lanes of NRF2/CAR signaling in the skeletal muscles of mice **(A)**; Relative protein expression of nuclear CAR **(B)**, nuclear NRF2 **(C)**, total CAR **(D)**, total NRF2 **(E)**, and total Keap1 **(F)**. Values are expressed as LSmean plus pooled SEM, *n*=3. ^*^Mean values were significantly different among groups (*p*<0.05). CAR, constitutive-androstane-receptor; Keap1, Kelch like ECH-associated protein 1; and NRF2, nuclear-factor-erythroid-2-related factor 2.

Compared with control, doxorubicin treatment led to decreased abundance of nuclear NRF2, as well as its downstream target CAR. This effect of doxorubicin was prevented by serine administration (Figures 4A–C). Doxorubicin-induced downregulation of Keap1, a regulator of NRF2 signaling, which was abolished by serine administration (Figure 4D). Protein abundance of total NRF2 and total CAR was not affected by doxorubicin, serine administration, or doxorubicin plus serine co-treatment (Figures 4E,F).

### Effects of Serine Administration on the Exercise Capacity of Mice

**Figure 5:**
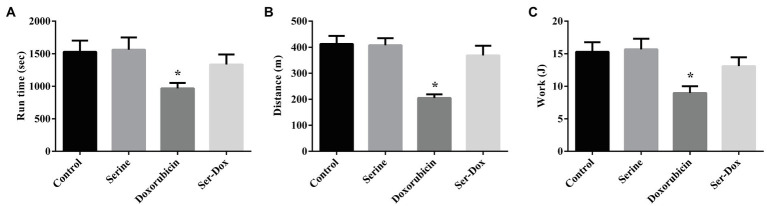
Effects of serine administration on the exercise capacity of mice. **(A)** Run time, **(B)** distance, and **(C)** excise performance were evaluated. Values are expressed as LSmean plus pooled SEM, *n*=8. ^*^Mean values were significantly different among groups (*p*<0.05).

To evaluate a functional role of serine administration on doxorubicin-induced skeletal muscle dysfunction, exercise capacity was examined by using the treadmill assay. As shown in Figure 5, mice exposed to doxorubicin had reduced exercise capacity as shown by shorter running time, shorter running distance, as well as a lower excise performance (Figures 5A–C), indicating a deleterious effect of doxorubicin on muscle function. Intriguingly, serine administration abolished the effect of doxorubicin on skeletal muscle, supporting a beneficial effect in muscle tissue of mice. Serine pre-administration had no effect on exercise capacity in mice.

## Discussion

In the present study, we found that serine administration reduced doxorubicin-induced oxidative damage, as evidenced by reduced protein, DNA, and lipid oxidation in skeletal muscle, as well as increased exercise capacity. This beneficial effect of serine was associated with reduced ROS accumulation, increased activity of the antioxidant enzymes, such as CAT, MnSOD, CuZnSOD, and increased intracellular GSH level, and upregulation of NRF2 signaling.

Doxorubicin is a chemotherapeutic drug for various malignances, including solid tumor and hemorrhage tumors. However, clinical application of doxorubicin leads to unexpected side effects on the muscle, therefore reducing quality of life and physical activity of patients. Oxidative damage is one of the major mediators that implicated in and contribute to muscle dysfunction in response to doxorubicin treatment. Nutritional strategy to reduce oxidative damage and restore muscle function is of great significance and attracts more and more attention. Our recent studies have shown that serine supplementation abrogates lipopolysaccharide-induced damage in animals and intestinal epithelial cells (Zhou et al., 2017b, 2018a). It remains unknown whether serine can protect mice against doxorubicin-induced skeleton muscle weakness.

In the present study, mice fed an AIN-93 diet supplemented with or without 1.5g/kgBW^−1^ day^−1^ serine were subjected to doxorubicin to induce oxidative damage in the skeleton muscle. Serine administration had no effect on feed intake and body weight gain during the whole experimental period (data not shown). In agreement with previous studies (Smuder et al., 2011; Min et al., 2015), doxorubicin injection resulted in enhanced oxidative damage of lipid, DNA, and proteins in skeleton muscle and reduced exercise capacity. Intriguingly, these alterations were not observed in serine-pretreated mice, indicating a preventive effect on oxidative damage in skeleton muscle.

To maintain intracellular homeostasis, the cells have evolved both enzymatic anti-oxidative systems, such as MnSOD, CuZnSOD, and CAT, as well as non-enzymatic anti-oxidative systems, including GSH and TRXN. In the present study, we found that doxorubicin exposure led to decreased activity of MnSOD, CuZnSOD, and CAT, indicating an impairment of enzymatic anti-oxidative capacity in response to doxorubicin. Interestingly, these effects of doxorubicin were abolished by serine pretreatment, indicating a regulatory effect of serine on anti-oxidative signaling. For the non-enzymatic antioxidant system, we found that GSH, but no TRXN level, were decreased by doxorubicin, thus implicating in oxidative damage in muscle tissues. GSH is one of the most abundant anti-oxidative tripeptides in the body (Ji et al., 1992). Our previous study has shown that glycine supplementation leads to increased GSH level in small intestine and serum, thus enhancing anti-oxidative capacity in piglet (Wang et al., 2014). Similar result was observed in skeletal muscles of doxorubicin-challenged mice. However, neither the content of cysteine (a key substrate for GSH synthesis) nor Gcl activity was affected by serine in the skeletal muscles of mice. Of note, we found that serine administration resulted in increased levels of serine, glycine, cysteine, and GSH, as well as an enhanced Gcl activity in the liver of doxorubicin-challenged mice. The liver is the body’s largest organ and the main source of GSH (DeLeve and Kaplowitz, 1991). The elevated production of GSH in the liver might contribute to an increased GSH content in the skeletal muscle, thus exerting a beneficial effect on oxidative damage. Availability of substrates and activity of enzymes implicated in GSH synthesis have been reported as critical factors for production of GSH in various conditions (Zhou et al., 2017a, 2018b), which might be one of the reasons for the elevated GSH in the skeletal muscle of mice (DeLeve and Kaplowitz, 1991). In addition to *de novo* synthesis of GSH, GRD can catalyze the GSSG to its reduced form and contribute to increased GSH level. Serine administration abrogated doxorubicin-induced downregulation of GRD, and led to increased GSH as observed in our study. These results indicated a regulatory effect of serine on intracellular redox homeostasis by enhancing GSH production (Meister and Anderson, 1983).

Mitochondria are the primary source for ROS production in skeletal muscle (Brand, 2010). Increased activities of electron-transport-complex of complex I, complex II–III, and complex IV, as well as decreased citrate synthase activity have been associated with elevated ROS level and oxidative damage in cells (Tarry-Adkins et al., 2016). In our study, we found that activity of complex I, instead of complexes II, III, and IV, was increased in response to doxorubicin administration, indicating that complex I was more sensitive to doxorubicin than other complexes. This result is agreement with previous study (Smuder et al., 2011). Importantly, the increased activity of complex I in the skeletal muscle and decreased activity of citrate synthase were significantly attenuated by serine administration. Additionally, doxorubicin-induced gene expression of *Hspd1*, a gene involved in stress response of the mitochondria, was partially prevented by serine. This is the first study showing that serine administration prevented the effect of doxorubicin on oxidative damage in muscle of mice.

The NRF2 is a master transcriptional factor that regulates the antioxidant defense systems. Under normal condition, the activity of NRF2 is restricted due to formation of the NRF2-Keap1 complex in cytosol (Chan et al., 2001). In response to oxidative stress, NRF2 is released and translocated to the nucleus, where it can trans-activate androstane receptor (CAR) signaling, which in turn, activates downstream anti-oxidative targets implicated in glutathione antioxidant system (Chan et al., 2001; He et al., 2017; Liu et al., 2018). To explore an involvement of the NRF2/CAR signaling pathway in doxorubicin-induced oxidative damage in skeletal muscle of mice, Western blot analysis was conducted to determine the protein abundance of Keap1, NRF2, and CAR. As expected, we found that doxorubicin exposure led to suppressed NRF2/CAR signaling, which was abrogated by serine administration, indicating a regulatory effect of serine and contribution to the beneficial effect.

## Conclusion

In conclusion, using doxorubicin-treated mice as a model of skeletal muscle weakness, we showed, for the first time, that serine administration prevented doxorubicin-induced oxidative damage by reducing ROS in the skeletal muscle of mice. This beneficial effect was associated with enhanced anti-oxidative capacity include both enzymatic and non-enzymatic systems in which NRF2-CAR signaling is implicated. Our study provides new insights on the functional role of serine in regulating intracellular redox homeostasis. Supplementation of serine might be a nutritional strategy to ameliorate skeletal muscle atrophy, a side effect observed in patients receiving doxorubicin treatment.

## Data Availability Statement

The raw data supporting the conclusions of this article will be made available by the authors, without undue reservation.

## Ethics Statement

The animal study was reviewed and approved by Committee of the Institute of Subtropical Agriculture, the Chinese Academy of Science.

## Author Contributions

JC and XZ performed the experiment, analyzed the data, and wrote the manuscript. HJ assisted the research and data analysis. XZ and ZW designed the study. ZW reviewed and edited the manuscript and had primary responsibility for final content. All authors contributed to the article and approved the submitted version.

## Funding

This study was supported by the National Natural Science Foundation of China (Nos. 31625025, 31572410, and 31272451), the “111” Project (B16044), National Key R&D Program of China (2018YFD0500405 and 2018YFD0501003), Natural Science Foundation of Hunan Province (2017JJ3373), and the earmarked fund for China Agriculture Research System (CARS-35).

## Conflict of Interest

The authors declare that the research was conducted in the absence of any commercial or financial relationships that could be construed as a potential conflict of interest.

## Publisher’s Note

All claims expressed in this article are solely those of the authors and do not necessarily represent those of their affiliated organizations, or those of the publisher, the editors and the reviewers. Any product that may be evaluated in this article, or claim that may be made by its manufacturer, is not guaranteed or endorsed by the publisher.

## Abbreviations

8-OHdG: 8-hydroxy-2'-deoxyguanosine
CAR: Constitutive-androstane-receptor
CAT: Catalase
CuZnSOD: Copper-zinc superoxide dismutase
Gcl: γ-glutamylcysteine ligase
GPX: Glutathione peroxidase
GRD: Glutathione reductase
GSH: Reduced glutathione
GSSG: Oxidized glutathione
GST: Glutathione S-transferase
Keap1: Kelch like ECH-associated protein 1
MDA: Malondialdehyde
MnSOD: Manganese superoxide dismutase
NRF2: Nuclear-factor-erythroid-2-related factor 2
ROS: Reactive oxygen species
