# Supplementary material for: Serine Supplementation Alleviates Doxorubicin-Induced Oxidative Damage in Skeletal Muscle of Mice
Source: Front Physiol. 2021 Sep 9;12:727093. doi: 10.3389/fphys.2021.727093 (PMC8458867; doi:10.3389/fphys.2021.727093)
Supplement: Supplementary file 1 [file Data_Sheet_1.docx]

Supplementary Material

**Serine supplementation alleviates doxorubicin-induced oxidative damage in skeletal muscle of mice**

**Jingqing Chen^1,2†^, Xihong Zhou^1,3†^, Hai Jia^1^, and Zhenlong Wu^1*^**

^1^ State Key Laboratory of Animal Nutrition, China Agricultural University, Beijing, 100193, China

^2^ Laboratory Animal Center of the Academy of Military Medical Sciences, Beijing, 100193, China

^3^ Key Laboratory of Agro-ecological Processes in Subtropical Region, Institute of Subtropical Agriculture, Chinese Academy of Sciences, Changsha 410125, China

**Supplementary Materials and Methods**

**Determination of amino acid content in the skeletal muscles and livers.**

Skeletal muscles from the upper hindlimb and liver samples were pretreated as described previously. The samples (50 mg) were incubated with 0.5 mL 10% sulfosalicylic acid, and maintained on ice for 10 min. The supernatant was collected by a centrifugation at 1,000 g at 4°C for 15 min, and then was filtered through 0.22-μm filters. The amino acid concentrations were analyzed by using an ion exchange amino acid analyzer (L8800, Hitachi, Tokyo, Japan).

**Supplementary Figures**


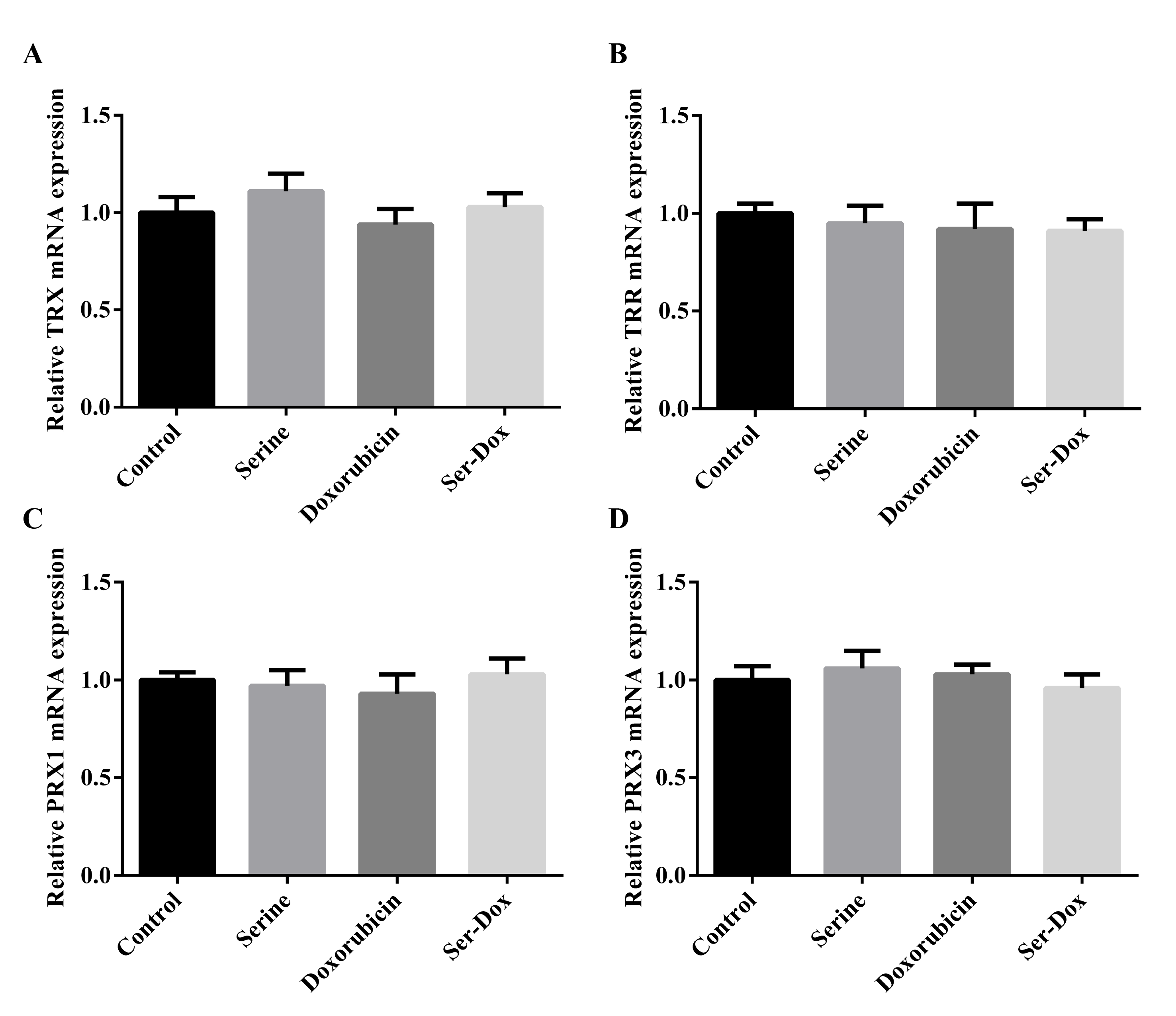


**Supplementary Figure 1.** Thioredoxin and peroxiredoxin antioxidant systems in the skeletal muscles of mice. Relative mRNA expression of TRX **(A)**, TRR **(B)**, PRX1 **(C)** and PRX3 **(D)** in the skeletal muscles. PRX, peroxiredoxin; TRX, thioredoxin 1; TRR, thioredoxin reductase 1. Values are expressed as LSmean plus pooled SEM, n=8.


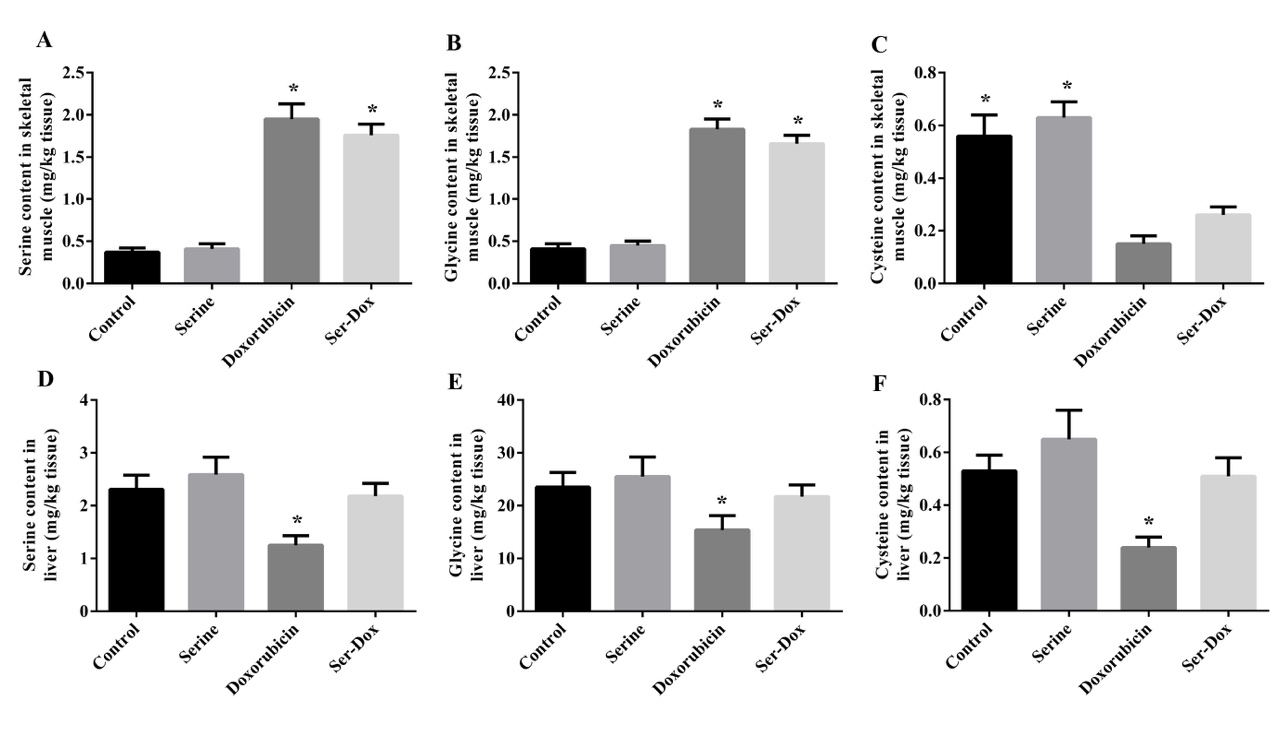


**Supplementary Figure 2.** Amino acids contents in the skeletal muscles and livers of mice. Serine **(A)**, glycine **(B)**, cysteine **(C)** contents in the skeletal muscles of mice. Serine **(D)**, glycine **(E)**, cysteine **(F)** contents in the livers of mice. Values are expressed as LSmean plus pooled SEM, n=8. ∗Mean values were signiﬁcantly different among groups ( *P* < 0.05).


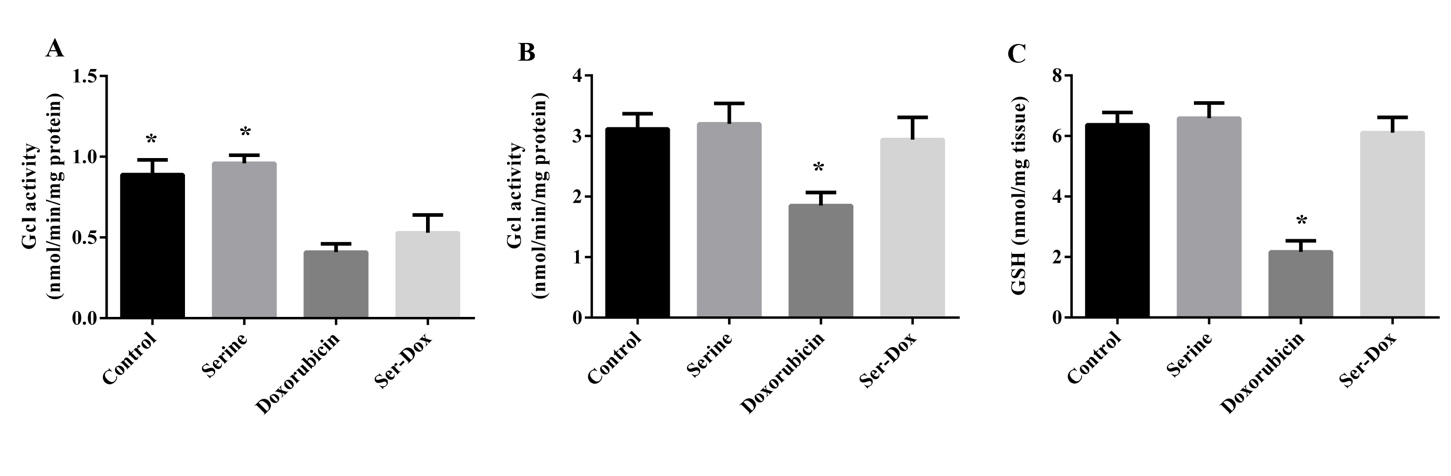


**Supplementary Figure 3.** Gcl activity or GSH content in the skeletal muscles and livers. Gcl activity in the skeletal muscles **(A)** and livers **(B)**. GSH content **(C)** in the livers of mice. Gcl, Glutamate cysteine ligase; GSH, reduced glutathione. Values are expressed as LSmean plus pooled SEM, n=8. ∗Mean values were signiﬁcantly different among groups ( *P* < 0.05).

**Supplementary Table 1.** Sequences of primers used for RT-qPCR.

| Gene | 5’-3’ Primer sequence |
| --- | --- |
| Prx1 | F: TGTCCCACGGAGATCATTGC |
|  | R: GGTGCGCTTGGGATCTGATA |
| Prx3 | F: TGGGCCACATGAACATCACA |
|  | R: GACACTCAGGTGCTTGACGA |
| TRR | F: CAATCTGAGCTGCCGAACAA |
|  | R: GGGATCTTTGGAGCCATTCA |
| TRX | F: GCGCTCCGCCCTATTTCTAT |
|  | R: TCACCATTTTGGCTGTTGCG |
| Hspd1 | F: TGATGTTGGCTGTGGATGCT |
|  | R: GACACCCTTTCTTCCAACCTTT |
| β-actin | F: TGTCCACCTTCCAGCAGATGT |
|  | R: AGCTCAGTAACAGTCCGCCTAGA |
